# Hairy roots: An untapped potential for production of plant products

**DOI:** 10.3389/fpls.2022.937095

**Published:** 2022-08-05

**Authors:** Kevin J. Morey, Christie A. M. Peebles

**Affiliations:** Department of Chemical and Biological Engineering, Colorado State University, Fort Collins, CO, United States

**Keywords:** hairy root, terpenoid indole alkaloid, metabolic engineering, bioreactor, *Catharanthus roseus*

## Abstract

While plants are an abundant source of valuable natural products, it is often challenging to produce those products for commercial application. Often organic synthesis is too expensive for a viable commercial product and the biosynthetic pathways are often so complex that transferring them to a microorganism is not trivial or feasible. For plants not suited to agricultural production of natural products, hairy root cultures offer an attractive option for a production platform which offers genetic and biochemical stability, fast growth, and a hormone free culture media. Advances in metabolic engineering and synthetic biology tools to engineer hairy roots along with bioreactor technology is to a point where commercial application of the technology will soon be realized. We discuss different applications of hairy roots. We also use a case study of the advancements in understanding of the terpenoid indole alkaloid pathway in *Catharanthus roseus* hairy roots to illustrate the advancements and challenges in pathway discovery and in pathway engineering.

## Introduction: Why hairy roots?

Plants are an abundant source of natural products which can be utilized in a wide variety of pharmaceutical and nutraceutical applications. For example, 25% of prescription drugs are derived directly or indirectly from plant compounds with a market value of greater than $30 billion. Interestingly, 66% of antimicrobial drugs and 52% of anticancer drugs are derived from plant sources (Oksman-Caldentey and Inze, 2004). Considering that only approximately 10% of higher plant species have had some sort of chemical characterization, there is a tremendous potential to discover new therapeutic compounds for the treatment of disease.

When a novel therapeutic lead is discovered, it is often challenging to supply enough of the compound for later phase clinical trials and for commercialization (Atanasov et al., 2015). Most of these natural products are classified as secondary metabolites with a complex chemical structure and are found at very low concentrations within the plant. Due to structural complexity, it can be a challenge both chemically and economically to produce these compounds through purely synthetic means. Thus, those in research and industry are reliant on biological systems to produce the natural product. For easily cultivatable plants, traditional agricultural practices can be utilized to produce the compound; however, the supply is subject to environmental factors (drought, flooding, or disease) that may adversely affect supply to the consumer. If the natural product is produced in slow-growing, hard-to-cultivate, or endangered plants, researchers must look for alternative routes to produce the compound. Often plant cell culture or tissue culture coupled with metabolic engineering is pursued as the best option.

Hairy root cultures are a particularly attractive option for production of many of these compounds (especially root produced compounds). Hairy roots are formed from the transfer of DNA (T-DNA) from the plant pathogen *Agrobacterium rhizogenes* (also called *Rhizobium rhizogenes*) into the plant host (Bahramnejad et al., 2019). The resulting hairy roots are fast growing, genetically and biochemically stable (Sun et al., 2017), easy to maintain, and grow in phytohormone free media. Due to the fact they are a differentiated tissue culture, they produce secondary metabolites at concentrations comparable to native roots. The production of a wide array of secondary metabolites have been explored in hairy roots. These include compounds such as alkaloids, anthocyanins (Barba-Espín et al., 2020), flavonoids, ginsenosides, phenolics, stilbenes, lignans, and terpenoids (Wawrosch and Zotchev, 2021). Hairy roots have also been used for the production of protein based products such as vaccines (Skarjinskaia et al., 2013; Massa et al., 2019), monoclonal antibodies (Donini and Marusic, 2019), and therapeutic proteins (Cardon et al., 2019; Zhang et al., 2019b).

Since T-DNA is transferred from *A. rhizogenes* into the nuclear genome of the plant, numerous metabolic engineering and synthetic biology tools have been developed to improve production of native products or to introduce heterologous products. To control gene expression, a variety of DNA parts have been developed including constitutive promoters, root specific promoters, inducible promoters, 5’UTRs, and terminators. CRISPR gene editing (Cai et al., 2015) and RNAi methodologies (Zhang et al., 2019a) have also been developed for use in hairy roots. These parts are utilized to improve expression of pathway genes and positive transcription factors or to knock down/out competing pathway genes or negative transcription factors. In this review, we will look at a variety of applications for hairy roots and will utilize a case study of recent research advancements to demonstrate the potential for and development of *Catharanthus roseus* hairy roots as a production system for the biosynthesis of terpenoid indole alkaloids (TIAs).

## Hairy roots: Research and applicational uses

### Synthetic biology, genome editing and genetic parts testing using hairy roots

Hairy roots have been useful for many applications in plant synthetic biology including genetic parts characterization, development of well-regulated gene circuitry, CRISPR/Cas9 based gene editing, and engineering stress tolerance. Some examples include the development of a gene stacking technique utilizing yeast to assemble DNA into plant transformation vectors. They then used their gene stacking technique to assemble a family of nine gene resistant clusters related to the Rps4-Rrs1 gene cluster involved in resistance to at least three known pathogens. Finally, as proof of concept they built a synthetic gene cluster fusing two of these clusters normally found on separate chromosomes and expressed it in soybean hairy roots to determine the impact on pathogen resistance (Shih et al., 2016). Hairy roots offer a convenient system to test the function of promoter elements without needing to regenerate a full plant. Tomato hairy roots (*Solanum lycopersicum*) were used to characterize the root cell type and root tissue specificity of 8 promoters using a promoter:nlsGFP-GUS reporter construct. Promoters with expression in the xylem, phloem, meristematic zone, cortex, lateral root cap and epidermis were characterized (Ron et al., 2014). Soybean hairy roots were used to test soybean cyst nematode (SCN)-inducible promoter motifs. Overlapping motif regions (OMRs) from the promoters of 18 soybean genes which are co-expressed during nematode infection were identified. Twenty three core motifs of 5–7 bp in length were confirmed to be nematode inducible when tested in soybean hairy roots (Liu et al., 2014). Hairy roots from the legume *Lotus japonicus* were used to test root expression of binary vectors that were assembled using a golden gate based, modular plasmid assembly kit for multigene expression, gene silencing and silencing rescue in plants (Binder et al., 2014).

Hairy roots can be used to develop and test gene circuits and synthetic biology tools. A memory switch based on the bacteria phage ϕC31 integration system was developed and tested in *Nicotiana benthamiana* hairy roots. The circuit was designed to control the on or off transcriptional state of two genes of interest by the alternative inversion of a central DNA regulatory element controlled by the ϕC31 integrase and its recombination directionality factor (Bernabe-Orts et al., 2020). A proof of concept gene editing study using CRISPR/Cas9 Technology was performed in Eucalyptus hairy roots targeting two wood-related genes: Cinnamoyl-CoA Reductase1 (CCR1), a key lignin biosynthetic gene and IAA9A an auxin dependent transcription factor of the Aux/IAA family. Most of the gene edits generated in this study resulted in truncated proteins (Dai et al., 2020). Tomato hairy roots were used by Jacobs and Martin to develop a protocol for high throughput CRISPR vector construction and subsequent characterizations of the CRISPR based modifications (Jacobs and Martin, 2016). CRISPR/Cas9 mediated mutagenesis of the steroid 16*α*-hydroxylase St16DOX gene in potato hairy roots resulted in the generation of α-solanine-free hairy roots (Nakayasu et al., 2018). Lastly, hairy roots are a great platform for testing hypotheses. Mutagenesis of the St16DOX gene reduced steroidal glycoalkaoids (SGAs) which taste bitter and can be toxic (Uluwaduge et al., 2018). The NAC (NAM, ATAF1/2 and CUC2) transcription factor family is involved in plant development and abiotic stress. Overexpression of GmNAC15 in soybean hairy roots enhances salt tolerance (Li et al., 2018).

### Studying the rhizosphere

#### Symbiotic root colonization and antimicrobial excretion

Hairy roots are an incredibly useful tool for the study of microrhizome interactions, both in understanding beneficial symbiotic root/microbial interactions and in pathogen defense through antimicrobial excretions by the roots. Bacteria of the *Candidatus Liberibacter* spp. are causal agents of citrus greening, potato zebra chip, and tomato vein greening diseases. Potato and tomato hairy roots were generated and infected with *C. Liberibacter* to enable high throughput identification of antimicrobials. This study identified six antimicrobial peptides, eight chemicals and two plant immune regulators which inhibit *C. Liberibacter* spp. in plants (Irigoyen et al., 2020). *Gmelina arborea* Roxb. is a medicinally important tree species which produces a phenylpropanoid glycoside antimicrobial called verbascoside. Hairy roots producing verbascoside were generated from *G. arborea*. Verbascoside is normally extracted from wild type roots but hairy roots offer an advantage when it comes to scaling up production of verbascoside (Dhakulkar et al., 2005). Shikonin derivatives are specialized lipophilic metabolites with antimicrobial properties which are secreted from the root epidermal cells of the medicinal plant *Lithospermum erythrorhizon*. Hairy Roots were generated from *L. erythrorhizon* and used as a model system to study lipid-soluble metabolite secretion (Tatsumi et al., 2016).

#### The role of master transcription factors controlling mycorrhizal signaling and colonization

Hairy roots have been used to study master transcription factors involved in mycorrhizal symbiosis. Arbuscular mycorrhiza (AM) fungi (Glomeromycotina fungi species) form a mutually beneficial symbiosis with land plants that improves nutrient acquisition from the soil in the plant in exchange for up to 20% of the host plant photosynthate. The AP2-Domain transcription factor WR15a was shown to be a master regulator of lipid biosynthesis and transfer during symbiosis in the legume *Medicago truncatula*. Both overexpression of WR15a and RNAi were performed in *M. truncatula* hairy roots to establish the role of WR15a in lipid biosynthesis and transfer as well as the subsequent role in establishing arbuscular mycorrhiza symbiosis (Jiang et al., 2018). Hairy roots from *M. truncatula* were also used to determine that a GRAS-domain transcription factor named RAM1 (Required for Arbuscular Mycorrhiza) is involved in mycorrhizal specific signaling. RAM1 was shown to regulate the expression of RAM2, a glycerol-3-phosphate acyl transferase that promotes cutin biosynthesis to enhance hyphopodia formation in arbuscular mycorrhiza fungi (Gobbato et al., 2012). Arbuscular mycorrhiza play a major role in providing the poorly accessible mineral nutrient, phosphate. Hairy roots derived from rice were used to establish the role of the master transcription factor Phosphate Starvation Response 2 (PHR2) in the enabling of arbuscular mycorrhiza symbiosis (Das et al., 2022).

### Phytoremediation using hairy roots

Phytoremediation is the use of plants to clean up environmental contaminants (Wei et al., 2021). Plant roots play an important role in phytoremediation in both uptake of the contaminant from the soil and in phytotransformation, the metabolic degradation of the contaminant. Hairy roots have been used as a phytoremediation tool either by taking advantage of the ability to scale up root tissue mass *via* hairy roots and utilizing natural phytoremediation ability or by expressing a transgene in the hairy roots that assists in phytotransformation. The ability of hairy roots derived from many different types of plants to assist in phytoremediation has been tested against a wide variety of environmental contaminants.

In terms of antibiotics, hairy root cultures of *Helianthus annuus* (sunflower) were shown to rapidly remove the antibiotics tetracycline and oxytetracycline from aqueous media, and the effect increased with culture age and the addition of the antioxidant ascorbic acid (Gujarathi et al., 2005). *Armoracia rusticana* (horseradish) hairy roots showed the ability to phytoremediate the pharmaceutical acetaminophen with 100% of the starting amount of acetaminophen being removed from the media during eight days (Kotyza et al., 2010).

In terms of industrial chemicals, *Lycopersicon esculentum* cv. Pera (tomato) hairy roots were shown to remove 100 mg/l of phenol from media in the presence of 5 mm hydrogen peroxide. This phytoremediation effect was attributed to basic peroxidase isoenzymes (González et al., 2006). Hairy root cultures of *Brassica napus* were used to phytoremediate 2,4-dichlorophenol (2,4-DCP), a common contaminant in industrial effluent. 97–98% of 2,4-DCP was removed from aqueous solutions containing 100–1,000 mg/l, in the presence of hydrogen peroxide concentrations ranging from 5 to 10 mm (Agostini et al., 2003). *Atropa belladonna*, a medicinal plant was transformed with a rabbit P450 2E1 transgene by *A. rhizogenes*. The hairy roots were exposed to trichloroethylene (TCE), a halocarbon commonly used as an industrial solvent. The P450 2E1 hairy roots showed enhanced TCE metabolism as measured by an increase in the presence of the TCE metabolites chloral and trichloroethanol (Banerjee et al., 2002). Endosulfan is a polychlorinated cyclodiene insecticide. Hairy roots from *Brassica napus*, *Raphanus sativus,* and *Capsicum annuum* were tested for their ability to remove endosulfan from the media, bioaccumulate, and biotransform. Hairy roots from *B. napus* and *C. annuum* removed 86% of Endosulfan from the media within 2 and 96 h. *R. sativus* hairy roots worked the best removing 91% of endosulfan within 6 h (Lucero et al., 2022).

There is a long history of using plants to remediate toxic metals, including Cu^2+^, Cr^6+^, Ni^2+^, Pb^2+^ and Zn^2+^ (Kumar et al., 1995). Horseradish hairy roots have also been used for arsenic phytoremediation. Hairy root tissues were able to remove as much as 75% of arsenic (in ranges from 5–60 μg/l) from the cultivation medium within 7 days (Kofronova et al., 2019). Hairy root cultures of *Brassica juncea* (brown mustard) and *Chenopodium amaranticolor* (a common laboratory plant used for viral tests) were tested for uranium phytoremediation ability. *B. juncea* hairy roots worked better for uranium removal. They could take up 2 to 4 times more uranium from the solutions than *C. amaranticolor*, at concentrations up to 5,000 mM. *B. juncea* hairy roots were saturated at 2000 mM concentration of uranium (Eapen et al., 2003).

### Producing and isolating plant secondary metabolites using hairy roots

The ability to scale up production levels and to genetically transform root tissue make hairy roots particularly useful in the production/purification of complex molecules derived from secondary metabolism, especially pharmaceuticals. The polyphenol curcumin is used in the management of oxidative and inflammatory conditions. Singh *et al.* used a synthetic biology approach for the production of curcumin and its glucoside in hairy roots of *Atropa belladonna*. They produced transformed hairy roots which heterologously expressed the curcumin biosynthetic pathway genes, diketide-CoA synthase (DCS) and curcumin synthase (CURS3), along with a glucosyltransferase gene (CaUGT2) to improve the yield of curcumin by ca. 2-fold (Singh et al., 2021a).

Tanshinones are a class of lipophilic phenanthrene compounds found in the roots of the traditional Chinese medicinal plant *Salvia miliorrhiza*. Tanshinones have been shown to exhibit antioxidant activity, anti-inflammatory activity, cardiovascular effects, and antitumor activity. They mainly accumulate in the roots, but the yield is very low, therefore attempts have been made to increase production using *S. miliorrhiza* hairy roots. A common approach to improving tanshinone production using *S. miliorrhiza* hairy roots is to overexpress transcription factors involved in the tanshinone biosynthetic pathway. Examples of this approach include overexpression of SmMYB9b (Zhang et al., 2017), SmMYB98 (Hao et al., 2020), SmbHLH10 (Xing et al., 2018b) and SmbHLH148 (Xing et al., 2018a).

Plant phenolic compounds have antioxidant, antimicrobial, UV protectant, and anti-inflammatory effects (Albuquerque et al., 2021). In addition to pharmaceutical and industrial uses, phenolic compounds can also serve as precursors for more complex secondary metabolites such as flavonoids and terpenoids. Some attempts to increase plant phenolics in hairy roots were part of an attempt to increase the end product yield for a desired secondary metabolite. The production of phenolic compounds has been explored in hairy roots either through production by phenolic pathway elicitation (chemical or hormonal) and/or through transgenic approaches.

Barba-Espín *et al*. developed hairy root cultures based on black carrots (*Daucus carota*), a major anthocyanin accumulator. When they exposed the cultures to ethephon, an ethylene (plant hormone) releasing compound, they substantially increased the anthocyanin content by up to 82% in one hairy root line and the production of hydroxycinnamic acids by >20% in another line (Barba-Espin et al., 2020). Elicitation using the plant hormone auxin in hairy roots derived from *Raphanus sativus* (L.; radish) resulted in an increase in the total amount of phenolics and flavonoids including the flavonoid quercetin which has antioxidant and anti-inflammatory effects (Balasubramanian et al., 2018). The flavone contents of hairy roots derived from *Scutellaria baicalensis,* an herb used in traditional Chinese medicine, were increased by 322% using a synthetic biology approach. *S. baicalensis* hairy roots were transformed with either Maize or Arabidopsis PAP1 (production of anthocyanin pigment) transcription factors which resulted in a larger yield of total flavonoid contents (Park et al., 2021). The bioactivity and total phenolic acid content of *Salvia miltiorrhiza* hairy roots was increased by transformation with transformation with RAS (rosmarinic acid synthase) and CYP98A14 (a cytochrome P450-dependent monooxygenase; Fu et al., 2020).

The untapped potential of hairy roots is now starting to be realized due to advances in fields like metabolomics, proteomics, transcriptomics, and genomics which provide the information and insights needed for applicational engineering of complex biological systems in hairy roots. Advancements in tools and techniques for genetic engineering such as *in vivo* gene editing using CRISPR, gene stacking techniques to create complex synthetic multi gene pathways or to transfer chromosomal fragments containing gene clusters and gene silencing techniques such as VIGS allow for applicational engineering which was not possible a few years ago. Metabolomic and genomic information allows for the selection of plant species and/or variants which will result in increases in product yield for applicational uses of hairy roots. The ability to transfer synthetic gene circuitry using techniques like gene stacking rather than just overexpress and/or knock-out one or two genes is invaluable for future hairy root genetic engineering. Transcriptomics studies in roots will allow for the selection of root, cell, and/or tissue specific promoters for use in synthetic circuits which will result in improved circuit expression compared to the classic CaMV 35S constitutive overexpression used for genetic engineering, especially when producing secondary metabolites that rely on tissue specific compartmentalization and/or transport to avoid toxicity issues. Transcriptomics/proteomics will be invaluable in identifying specific transporters for the metabolite of interest. Another improvement in the field of hairy roots is the use of mathematical modeling to improve hairy root cultivation conditions and to maximize elicitor combinations and/or timing. Using metabolomics data to model biosynthetic pathways will also be invaluable in tapping the production potential of hairy roots, especially in identifying rate limiting enzymes and key secondary metabolite branchpoints. There has also been valuable empirical work performed in the recent years which has improved the bioreactors needed for scaling up hairy roots for plant product production.

## Case study: Recent research advancements influencing the engineering of *Catharanthus roseus* hairy roots for production of terpenoid indole alkaloids

*Catharanthus roseus* (Madagascar periwinkle) produces over 130 terpenoid indole alkaloids (TIAs). Pharmaceutically important TIAs which have been isolated from *C. roseus* include vinblastine and vincristine which have been used as anticancer drugs and ajmalicine and serpentine which are anti-hypertensive and anti-neuro-inflammatory agents (Almagro et al., 2015). TIAs accumulate at low levels *in planta* resulting in low extraction yields. For example, in one study extraction of vinblastine from *C. roseus* leaf tissue only yielded ca. 0.00025% of the dry leaf weight (Ishikawa et al., 2009). In a study that investigated the variability of TIA precursors vindoline and catharanthine. They found that 50 days after sowing the seeds that vindoline production ranged from 241 μg/gdw to 2082 μg/gdw and catharanthine production ranged from 273 μg/gdw to 2,903 μg/gdw (Chung et al., 2011). Commercial production currently comes from the harvesting of catharanthine and vindoline from field grown plants followed by the semi-synthesis of vinblastine and vincristine. Despite this production route, these two chemicals often show up on the FDA drug shortage list due to supply chain problems (Kaiser, 2011). There is also an inability to industrially mass produce TIAs *via* chemical synthesis.

These shortfalls have led to research into increasing the production of TIAs through other routes. To date, *S. cerevisiae* has been engineered to produce strictosidine at a level of 530 μg/L after an unreported time of cultivation (Brown et al., 2015). Several groups have also worked to produce vindoline from tabersonine (Mistry et al., 2022) this strategy still relies on plant produced tabersonine. A hybrid production system using plant cell or tissue culture to produce tabersonine while yeast is used to produce vindoline could be one interesting route to improve commercial availability of vinblastine and vincristine. Plant cell or tissue culture alone offer another route to the production of TIAs (Table 1). Plant cell suspension cultures need hormones to maintain growth and only produce TIAs while elicited. Plant cell cultures are notorious for genetic instability resulting in loss of production of secondary metabolites. Hairy root tissue cultures offer a way to improve TIA production while growing the biomass in an enclosed and well controlled bioreactor system. Recent efforts in engineering *C. roseus* hairy roots have resulted in the production of ~1,500 μg/gdw of catharanthine and ~ 6,000 μg/gdw of aspidosperma alkaloids (tabersonine, hörhammercine and lochnericine; Sun and Peebles, 2016). These concentrations are within the same order of magnitude that is produced in whole plants on a dry weight basis, but hairy roots grow significantly faster than whole plants and are not limited by seasonal production and by environmental stress that hamper growth and production. The overexpression of tabersonine 16-hydroxylase and 16-hydroxytabersonine-O-methyltransferase in *C. roseus* hairy roots demonstrated the potential toward redirecting aspidosperma alkaloids toward vindoline production in hairy roots (Sun et al., 2018). As an illustration of the utility of using hairy roots as a metabolic engineering platform, we give examples of recent advancements (ca. the last 5 years) in TIA biosynthetic pathway elucidation and expression of TIAs in *C. roseus* hairy roots, including other *C. roseus* work which may be applied toward hairy root metabolic engineering for TIA biosynthesis.

**Table 1:** Comparison of advantages and disadvantages of plant culture types.

	Whole plant	Cell suspension culture	Hairy roots
Secondary metabolite production		Often only with elicitation	Comparable to whole plants (esp. roots) without elicitation
Growth rate	Slow	Fast (days)	Fast (days)
Genetic and biochemical stability	Stable	Unstable	Stable (demonstrated over 10 years stability)
Metabolic engineering	Yes, but can be difficult in some cases	Yes	Yes
Scale up potential	Restricted to agricultural lands, climate zones, seasons, and impacted by environmental stressors	Yes	Yes
Cost	Cheap	Expensive	Expensive

### TIA biosynthetic branch pathway elucidation

**Figure 1:**
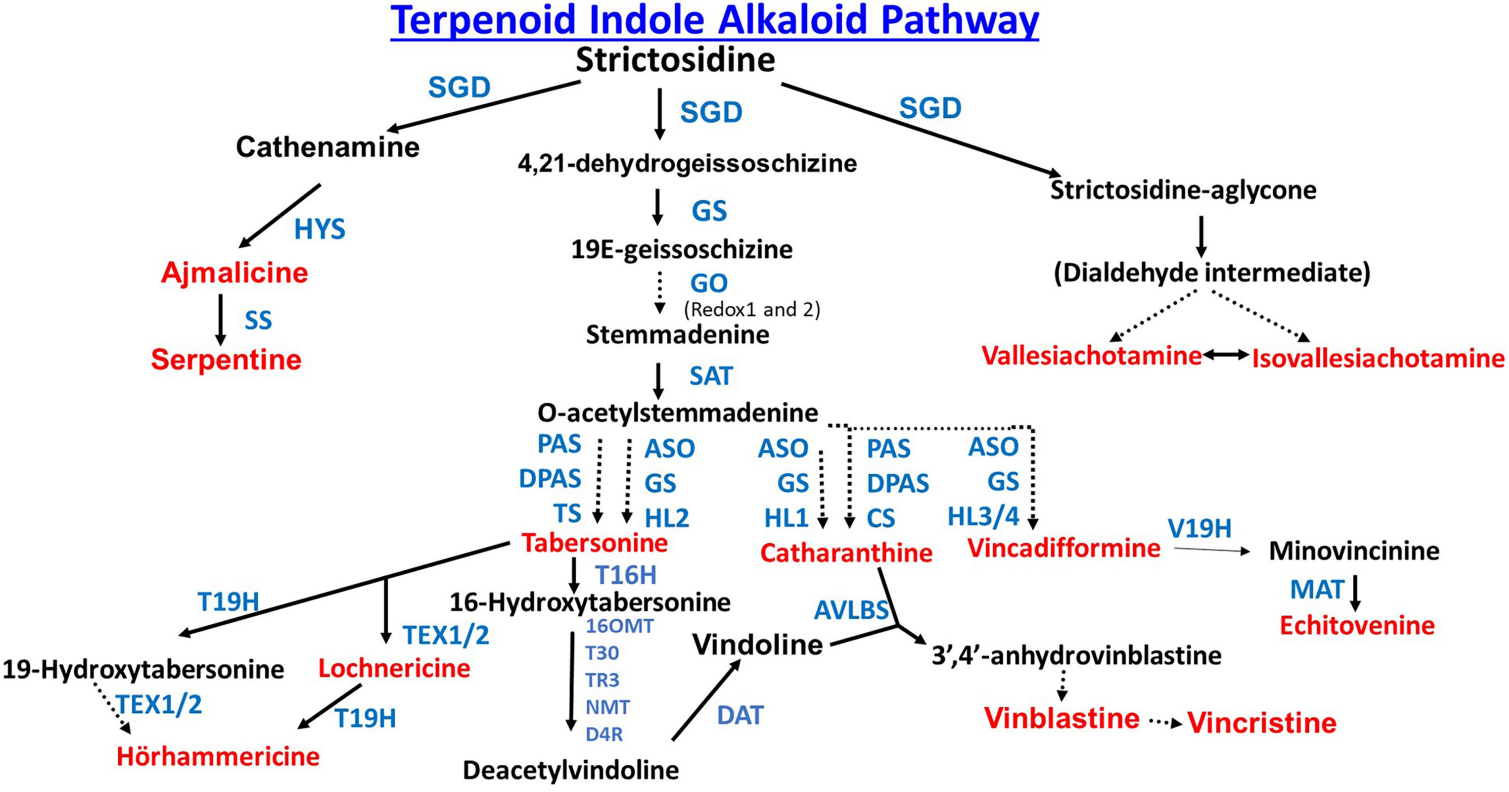
Biosynthesis of strictosidine, the common precursor of all terpenoid indole alkaloids. Enzyme abbreviations are as follows: AACT (acetoacetyl-CoA thiolase), AS (anthranilate synthase), CPR (cytochrome P450 reductase), CYP72A1v3 (cytochrome P450 reductase), DXR (1-deoxy-D-xylulose-5-phosphate reductoisomerase), DXS (1-deoxy-d-xylulose-5-phosphate synthase), G10H (geraniol 10-hydroxylase), GES (geraniol synthase), GPPS (geranyl diphosphate synthase), HMGR (hydroxymethyglutaryl-CoA reductase), HMGS (hydroxymethyglutaryl-CoA synthase), IGPS (indole-3-glycerol phosphate synthase), IPI (isopentenyl diphosphate isomerase), MVD (mevalonate 5-diphosphate decarboxylase), MVK (mevalonate kinase), PAT (phosphoribosylanthranilate transferase), PAI (phosphoribosyl anthranilate isomerase), PMK (mevalonate 5-phosphate kinase), SLS (secologanin synthase), STR (strictosidine synthase), TDC (tryptophan decarboxylase), and TS (tryptophan synthase).

Production of TIAs involves the convergence of two pathways (Figure 1). The first pathway, the indole pathway starts with the methylerythritol dicyclophosphate (MEP) or mevalonate (MVA) pathways both of which produce isopentenyl pyrophosphate (IPP). The MVA pathway is cytosolically localized, while the MEP pathway is localized in the chloroplast. The MEP pathway forms IPP from glycerol 3-phosphate (G3P) and pyruvate in 7 enzymatic steps (Zeng and Dehesh, 2021). The MVA pathway forms IPP from acetyl-CoA in 6 enzymatic steps (Miziorko, 2011). It is interesting to note in the legume *Medicago truncatula*, a key regulatory MVA pathway enzyme, 3-hydroxy 3-methylglutaryl CoA reductase 1 (HMGR1) is directly involved in the signaling pathway that transduces endosymbiotic microbial signals (Venkateshwaran et al., 2015). IPP is converted to the monoterpene secologanin in 9 enzymatic steps (Kai et al., 2015).

**Figure 2:**
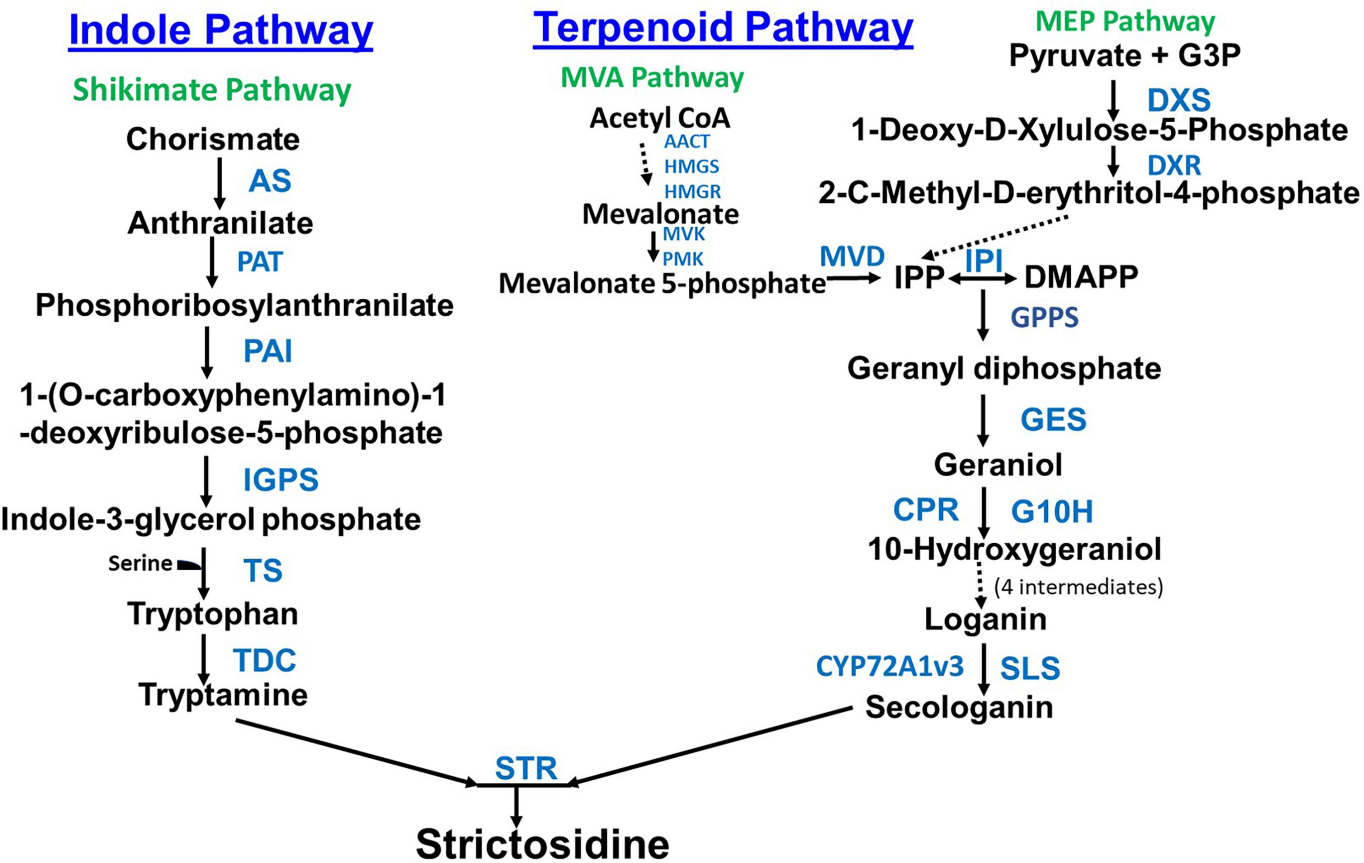
Biosynthesis of terpenoid indole alkaloids starting from strictosidine. Enzyme abbreviations are as follows: 16OMT (16-hydroxytabersonine 16-O-methyltransferase), ASO (O-acetylstemmadenine oxidase), AVLBS (anhydrovinblastine synthase), CS (catharanthine synthase), D4R (desacetoxyvindoline 4-reductase), DAT (deacetylvindoline 4-Oacetyltransferase), DPAS (dihydroprecondylocarpine synthase), GO (geissoschizine oxidase), GS (geissoschizine synthase), HL1 (α/β hydrolase 1), HL2 (*α*/*β* hydrolase 2), HYS (heteroyohimbine synthase), NMT (N-methyltransferase), PAS (precondylocarpine acetate synthase), SAT (stemmadenine-O-acetyltransferase), SGD (strictosidine β-D-glucosidase), SS (serpentine synthase), T16H (tabersonine 16-hydroxylase), T19H (tabersonine 19-hydroxylase), T3O (tabersonine 3-oxidase), TEX1/2 (tabersonine 6,7-epoxidase), TR3 (tabersonine 3-reductase), and TS (tabersonine synthase).

The second pathway, the terpenoid pathway starts with the shikimate pathway, a seven step pathway which forms chorismate from phosphoenolpyruvate and erythrose-4-phosphate (Maeda and Dudareva, 2012). Chorismate is then converted to tryptamine in seven steps (Figure 1). Secologanin from the terpenoid pathway and tryptamine from the indole pathway are then joined to form the TIA strictosidine through the actions of the enzyme Strictosidine synthase (STR). Strictosidine is converted to Cathenamine which is a precursor to the monomeric indole alkaloids (MIA) ajmalicine and serpentine and bisindole alkaloids (vinblastine and vincristine; Figure 2). Throughout the TIA pathway there are highly malleable intermediates and by-products which leads to a high TIA end product diversity (Qu et al., 2019). Engineering TIA biosynthesis is a complex undertaking due to the number of TIA metabolites, compartmentalization of different biosynthetic pathway branches, and both cell type and tissue specificity (reviewed in Kulagina et al., 2022; Figure 2 is especially good). In leaves and stems, TIA biosynthesis occurs in different cell types starting from internal phloem-associated parenchyma cells (IPAP cells) through epidermal cells (ECs) to both idioblast cells (ICs) and laticifer cells (LCs; Yamamoto et al., 2016; Qu et al., 2019).

#### Recent examples of research using biochemistry and molecular biology for the elucidation and discovery of TIA enzymes and pathways

For decades, the discovery and validation of plant natural product pathway genes was a slow and arduous process. This has dramatically changed in the past decade with the advancement of functional genomics tools. The field has seen a reduction in cost of next generation sequencing and an explosion of transcriptomic studies applied to plants like *C. roseus*. Techniques such as virus induced gene silencing (VIGS) and transient agrobacterium-based protein expression in *Nicotiana benthamiana* have been used along with mass spectrometry to validate potential gene candidates. VIGS is a technique which utilizes post transcriptional gene silencing (PTGS) to target the function of genes of interest (Bekele et al., 2019). VIGS can be complemented with overexpression to elucidate the contribution of key enzymes in the TIA biosynthesis pathway. Examples of utilizing transcriptomics, gene overexpression and VIGS to complete our knowledge of the TIA biosynthetic pathway are described below.

Qu *et al*. characterized a *C. roseus* geissoschizine synthase (GS) mutant that accumulates high levels of ajmalicine at the expense of catharanthine and vindoline. GS is involved in the production of 19E-geissoschizine, a precursor of catharanthine and tabersonine (Figure 2). VIGS was then used to demonstrate the role of GS in controlling metabolic flux in the formation of TIAs (Qu et al., 2018b). Qu et al. functionally characterized six genes as well as a seventh enzyme reaction required for the conversion of 19E-geissoschizine to tabersonine and catharanthine. They further described the role of stemmadenine-O-acetylation in providing necessary reactive substrates for the formation of the iboga and aspidosperma class of alkaloids (Qu et al., 2018a). Yamamoto *et al*. used an improved VIGS methodology to identify a previously unknown TIA biosynthetic enzyme, serpentine synthase (SS) in *C. roseus* (Figure 2). SS is an ER localized cytochrome P450 (Yamamoto et al., 2021). SS is a homolog of the cytochrome P450, alstonine synthase (AS) that produces the stereoisomer of serpentine, alstonine (Dang et al., 2018).

Strictosidine b-D-glucosidase (SGD) acts at the key step just after tryptamine joins with secologanin to form strictosidine (Figure 2). SGD deglycosylates strictosidine which is a key activation point for the downstream TIA pathway. Transcriptomics led to the discovery of an alternate splicing of SGD which results in a shorter isoform shSGD. shSGD negatively interacts with SGD inhibiting its activity, adding another control layer in the TIA biosynthesis pathway (Carqueijeiro et al., 2021).

Two candidate enzymes were identified using RNA sequencing (RNA-seq) data to further fill in the vinblastine biosynthetic pathway, an oxidase precondylocarpine acetate synthase (PAS) and a reductase dihydroprecondylocarpine synthase (DPAS) that isomerize stemmadenine acetate into dihydroprecondylocarpine acetate (Figure 2). This work also led to the discovery of the catalytic function of two other hydrolase enzymes, catharanthine synthase (CS) and tabersonine synthase (TS) that convert stemmadenine acetate to catharanthine and tabersonine, respectively (Caputi et al., 2018 Figure 2). *In vitro* and *in vivo* studies found that assembly of dimeric TIAs requires O-acetylstemmadenine oxidase (ASO) and a dual function geissoschizine synthase (GS) which reduces cathenamine to form geissoschizine, and reduces the ASO produced O-acetylstemmadenine intermediate product to form an intermediate that is converted by four separate hydrolases to catharanthine, tabersonine, or vincadifformine (Qu et al., 2019; Figure 2).

Using transcriptome databases, Miller *et al*. identified and characterized two cytochrome P450 reductases (CPRs) that were involved in the pathway divergence in *C. roseus* and *Camptotheca acuminata* which leads to vinblastine and vincristine production in *C. roseus* and camptothecin production in *C. acuminata*. The two *C. acuminata* P450s, CYP72A564 and CYP72A565, can utilize both loganic acid and loganin to generate secologanic acid and secologanin. In contrast the *C. roseus* CYP72A1v3 metabolizes loganin but not loganic acid (Miller et al., 2021; Figure 1).

While new techniques have pushed pathway discoveries forward, it is important to not forget proven pathway elucidation methods such as feeding studies and metabolite analysis. Feeding studies using catharanthine showed an increase in the biosynthesis of the MIAs vallesiachotamine (shown to have antiproliferative effects on human melanoma cells in-vitro (Soares et al., 2012)) and isovallesiachotamine in *C. roseus* Cambial meristematic cells. There was an observed upregulation of specific TIA pathway enzymes and transcription factors, including STR, Strictosidine b-D-glucosidase (SGD), and ORCA3 (transcription factor octadecanoid-responsive Catharanthus AP2/ERF-domain). Based on the genes which were upregulated, the outline for a biosynthetic pathway to make vallesiachotamine and isovallesiachotamine from strictosidine was proposed (Jianhua Zhua et al., 2018; Figure 2). Similarly, lochnericine is a major MIA derived from the stereoselective C6,C7-epoxidation of tabersonine in *C. roseus* roots. Combining gene correlation studies, functional assays, and VIGS led to the identification of two conserved P450s that efficiently catalyze the C6,C7-epoxidation of tabersonine. The P450s were named tabersonine 6,7-epoxidase isoforms 1 and 2 (TEX1 and TEX2; Carqueijeiro et al., 2018a; Figure 2). Echitovenine, is the major O-acetylated MIA in *C. roseus* roots. Williams et al. used transcriptomics to identify an enzyme, vincadifformine 19-hydroxylase (V19H) which converts vincadifformine to minovincinine (Figure 2). A three step pathway for the biosynthesis of echitovenine, which parallels the biosynthesis of a major root MIA, hörhammericine was characterized. The pathway starts with the conversion of O-acetylstemmadenine to vincadifformine. Vincadifformine is then converted to minovincinine by V19H and minovincinine is then converted to echitovenine by minovincinine-O-acetyltransferase (MAT; Williams et al., 2019) Carqueijeiro et al. used a transcriptomics approach to identify an O-acetyltransferase counterpart of MAT that is specific for 19-tabersonine. They were able to identify O-acetyltransferase candidates based on an expression correlation with the enzyme tabersonine 19-hydroxylase (T19H) and localization in small genomic clusters. A single candidate gene which identified which was able to acetylate minovincinine and hörhammericine, 19-hydroxytabersonine derivatives found in roots. The gene was named tabersonine derivative 19-O-acetyltransferase (TAT; Carqueijeiro et al., 2018b).

Alkaloid profiling of tissues from *C. roseus* seedlings was used to suggest the following initial biosynthetic pathway from tabersonine to vindoline: tabersonine → 16-hydroxytabersonine → 16-methoxytabersonine → 3-hydroxy-16-methoxy-2,3-dihydrotabersonine → desacetoxyvindoline → deacetylvindoline → vindoline. Vindoline synthesis was enhanced by light but light wasn’t essential. Tabersonine biosynthesis was found to occur in all plant tissues whereas the last 5 steps in vindoline biosynthesis were restricted to aerial tissues (Deluca et al., 1986). The enzymes needed to synthesize vindoline from tabersonine and when they were originally characterized is as follows. Tabersonine to 16-hydroxytabersonine requires a cytochrome P450, tabersonine 16-hydroxylase (T16H; Schröder et al., 1999). 16-hydroxytabersonine to 16-methoxytabersonine requires an O-methyltransferase (16OMT; Levac et al., 2008). 16-methoxytabersonine to 3-hydroxy-16-methoxy-2,3-dihydrotabersonine requires tabersonine 3-oxygenase (T3O) and tabersonine 3-reductase (T3R; Qu et al., 2015). 3-hydroxy-16-methoxy-2,3-dihydrotabersonine to desacetoxyvindoline requires 3-hydroxy-16-methoxy-2,3-dihydrotabersonine N-methyltransferase (NMT; Deluca et al., 1987). Desacetoxyvindoline to Deacetylvindoline requires desacetoxyvindoline-4-hydroxylase (D4H; Vazquez-Flota et al., 1997). Deacetylvindoline to Vindoline requires deacetylvindoline-4-O-acetyltransferase (DAT; St-Pierre et al., 1998).

#### Recent research involving TIA tissue specificity and compartmentalization

TIA biosynthetic enzymes occurring at pathway branches are expressed in a cell type dependent and organ specific manner (reviewed in Watkins and Facchini, 2022) and (Kulagina et al., 2022). A transcriptomics analysis was performed on five *C. roseus* tissues: stem, hairy root, flower, immature and mature leaves. The analysis focused on six key TIA pathway genes SLS, SGD, STR1, OMT, DAT and G8H. The highest expression levels for these genes were found in the root, flowers, and mature leaves (Ramadan et al., 2020).

There is also evidence of epigenetic regulation of TIA biosynthesis. The developmental methylome of *C. roseus* was examined. Tissue specific expression was associated with specific methylation signatures. Some genes encoding key enzymatic steps from the TIA pathway were found to be simultaneously differentially expressed and methylated. The transcription factor ORCA2 displayed hypermethylation in two differentially methylated regions (DMRs) which corresponded to a higher expression in roots vs. mature leaves (Duge de Bernonville et al., 2020).

#### Transcriptional control of the TIA biosynthetic pathway

The TIA pathway is controlled by a group of positive and negative transcriptional regulators which respond to a variety of signals (reviewed in Liu et al., 2017a). Previously discovered positive TIA transcriptional regulators include ORCAs (octadecanoid-responsive Catharanthus AP2/ERF-domain proteins; Memelink et al., 2001), BIS (bHLH Iridoid Synthesis; Patra et al., 2018), CrWRKY1 (Suttipanta et al., 2011), and CrGATA1 (Liu et al., 2019). These transcription factors respond to different environmental signals. ORCAs and BIS are modulated by methyl-jasmonate and ethylene. WRKY transcription factors are key regulators in defense responses. GATA transcription factors are involved in gene induction by light. Negative TIA transcriptional regulators include the Cys2/His2-type (transcription factor IIIA-type) zinc finger protein family ZCT1, ZCT2 and ZCT3. The ZCTs have been shown to directly bind to the promoters for two enzymes of the TIA biosynthetic pathway, tryptophan decarboxylase (TDC) and strictosidine synthase (STR; Memelink and Gantet, 2007). Other negative transcriptional regulators are G-Box Binding Factors (GBFs; Sibéril et al., 2001), Jasmonate Zim-Domain Proteins (JAZs; Patra et al., 2013), Repressor of MYC2 Targets (RMT; Patra et al., 2018) and *C. roseus* Phytochrome Interacting Factor (CrPIF1; Liu et al., 2019).

To add to the level of complexity, some of these transcription factors are activated post transcriptionally through mitogen-activated protein kinases (MAPK). This activation is most likely through the phosphorylation of CrMYC2 and ORCAs transcription factors (Paul et al., 2017). In *C. roseus* leaves, a heat activated MAPK, CrMAPK3 upregulates the expression of the upstream TIA biosynthetic enzymes TDC, STR and the downstream biosynthetic enzymes D4H, and DAT (Raina et al., 2013).

##### Recent discoveries (2017–2021) involving positive TIA biosynthesis regulators

The complex regulation of the TIA pathway is demonstrated by the following studies. These discoveries are enabled by a number of functional genetics approaches including transcriptomics, overexpression studies, and knock down studies. A basic helix–loop–helix factor (bHLH) CrMYC2 positively regulates TIA biosynthesis. The activity of CrMYC2 is regulated by repressors, such as the bZIP factors CrGBF1 and CrGBF2. Overexpression of CrMYC2 induces CrGBF expression reducing TIA accumulation. CrGBFs repress activation by competitively binding to the T/G-box in the TIA biosynthesis pathway promoters and/or protein–protein interaction with CrMYC2 that forms a non-DNA binding complex that prevents CrMYC2 from binding (Sui et al., 2018).

*Catharanthus roseus* transcriptome data for TIA pathway branch specific transcription factors (TFs) identified additional members of the known BIS and ORCA TFs. Functional analysis of the ORCA TF paralogs suggested sub-functionalization in terms of gene-specific regulation and uncovered synergistic activity with the jasmonate MYC2 response regulator (Colinas et al., 2021). Genomic analysis of BIS transcription factors BIS1 and BIS2 revealed a third family member BIS3. Characterization of BIS3 showed it was highly expressed in roots and induced by methyl jasmonate. BIS3 activates the promoters of terpenoid pathway genes. The genes activated by BIS3 are GES, G10H (Figure 1), and the genes involved in the conversion of 10-hydroxygeraniol to loganin; 8-hydroxygeraniol oxidoreductase (8HGO), iridoid synthase (IS), 7-deoxyloganetic acid glucosyl transferase (7-DLGT), and 7-deoxyloganic acid hydroxylase (7DLH), but not iridoid oxidase (IO; Singh et al., 2021b).

The jasmonic acid responsive AP2/ERF transcription factor, ORCA3 plays a key role in TIA biosynthesis and is also regulated by CrMYC2. ORCA3 was shown to be part of a gene cluster with previously uncharacterized transcription factors ORCA4 and ORCA5. ORCA3, 4 and 5 were all shown to strongly activate the STR promoter. All three transcription factors also activate the Terpenoid pathway *via* the CPR promoter and the indole pathway via the TDC promoter (Figure 1) with varying strengths. This ORCA gene cluster is differentially regulated. ORCA4 functionally overlaps with ORCA3 but it also modulates an additional set of TIA genes. ORCA4 (but not ORCA3) overexpression resulted in dramatic increase of TIA accumulation in *C. roseus* hairy roots. Co-expression of ORCA3, 4 and 5 with the MAPK kinase CrMPKK1 resulted in an increase in reporter gene expression (Paul et al., 2017). A closer examination of this ORCA3, ORCA4, and ORCA5 gene cluster showed that there were two additional AP2/ERFs, the previously characterized ORCA2 and a newly identified member designated as ORCA6. ORCA6 heterologously activated the STR promoter in tobacco cells. STR promoter activation by ORCA6 was higher if co-expressed with CrMPKK1. Overexpression of ORCA6 in *C. roseus* flower petals vs. an empty vector control led to an increase in tabersonine levels but no increase in ajmalicine, catharanthine or vindoline (Singh et al., 2020b).

RNA seq identified an AP2/ERF transcription factor, CrERF5 which responds to both ethylene and JA signals. Overexpressing CrERF5 in *C. roseus* petals caused a significant increase of the expression levels of key MIA biosynthesis genes in both the upstream and downstream pathways. This led to an increase of anhydrovinblastine, vinblastine, ajmalicine, vindoline, and catharanthine (Pan et al., 2019).

##### Recent discoveries (2017–2021) involving negative TIA biosynthesis regulators

An AP2/ERF transcription factor, CR1 was identified using RNA-seq data from methyl jasmonate (MeJA)-treated *C. roseus*. VIGS targeting of CR1 led to an increase in TIA biosynthetic gene expression and an increase in vindoline and serpentine levels (Liu et al., 2017b). An miRNAome analysis of *C. roseus* identified 181 conserved and 173 novel miRNAs in *C. roseus* seedlings. A subset of the miRNAs was shown to be differentially regulated in response to MeJA. The phytohormone auxin, indole acetic acid, represses the expression of key TIA pathway genes in *C. roseus* seedlings. There is an auxin responsive transcription factor (ARF), CrARF16, which binds to the promoters of key TIA pathway genes and repress their expression. CrARF16 is regulated by miRNAs (Shen et al., 2017).

In an attempt to increase TIA levels, *C. roseus* hairy roots were elicited with MeJA, while simultaneously silencing the expression of the transcriptional repressor ZCT1 using an estrogen inducible Zct1 hairpin for activating RNAi. Silencing Zct1 was not sufficient to increase TIA production or the expression of the TIA biosynthetic genes (Rizvi et al., 2016). A follow up study attempted to silence all three negative transcriptional regulators of TIA genes, ZCT1, ZCT2 and ZCT3. An RNAi construct targeting all three repressors under the control of a beta-estradiol inducible promoter was established in transgenic *C. roseus* hairy root lines. Out of eight TIA biosynthetic genes analyzed, seven (CPR, LAMT, TDC, STR, 16OMT, D4H and DAT) exhibited an unexpected decrease when the ZCTs were repressed. The one exception was tabersonine 19-hydroxylase (T19H). T19H differs from the other biosynthetic genes in that it shunts metabolites away from vindoline production and the other seven biosynthetic enzymes shunt metabolites towards vindoline production. It was hypothesized that ZCT regulation of the seven TIA biosynthetic genes tested is likely to occur indirectly, possibly by the ZCTs turning off expression of a negative transcriptional regulator of some TIA genes. A known negative transcriptional regulator, GBF1 shows a strong negative correlation with ZCT transcript levels (Li and Gibson, 2021). This demonstrates the complexity of TIA biosynthesis regulation wherein negative regulators are subject to negative regulation.

An antisense knockdown study of ZCT1, ZCT2 and ZCT3 used LNA GapmeRs transfection into protoplasts of *C. roseus* photomixotrophic cell suspensions to develop knockdown cell lines. The ZCT3 knockdown line Z3G line had vindoline levels at 0.038 ± 0.001 mg/g dry wt., catharanthine at 0.165 ± 0.008 mg/g dry wt. and vinblastine 0.0036 ± 0.0003 mg/g dry wt. The cell line showed multifold increase in the gene expression of D4H and DAT which catalyze the last two steps in vindoline synthesis. In the TIA pathway TDC and STR were upregulated. The ZCT repressors are known to directly bind the promoters to these genes. SGD, SLS, T16H, and the apoplastic peroxidase gene (PRX) were all upregulated in the Z3G line. The CrPRX gene is involved in the regulation of TIA pathway genes and regulators (Verma et al., 2021). The positive results of the ZCT3 knockdown in this study versus the pathway repression seen in the hairy root ZCT1, ZCT2, and ZCT3 knockdown may be related to the tissue types used, i.e., chlorophyllous cells vs. root tissue.

### Elicitors of TIA biosynthesis

TIAs are secondary metabolites which play a major role in a plants response to environmental stresses including pathogen defense (Long et al., 2018), herbivory (Duge de Bernonville et al., 2017) oxidative stress including heavy metal exposure (Dubey et al., 2014; Matsuura and Fett-Neto, 2014) and light stress (Jaiswal and Agrawal, 2018). One strategy to increase the production of TIAs is either to expose the plant material to a chemical or environmental stress inducer to elicit TIA production or directly use the plant hormones (phytohormones) induced by the environmental stresses. The following are recent examples of *C. roseus* research involving TIA elicitors.

#### Pathogen defense elicitation

Chitin is a major cell wall constituent of fungus (Liaqat and Eltem, 2018) Chitooligosaccharides derived from chitin are elicitors of innate immune defense against fungal pathogens. Treatment of *C. roseus* leaves using 0.1 mg/ml of a 3 kDa molecular weight commercially available chitooligosaccharide extraction led to an increase of the vinblastine precursors vindoline and catharanthine of 60.68 and 141.54% (Tang et al., 2022). In *C. roseus* cambial meristematic cell cultures adding 25 mg/l mycelium elicitor from the fungus *Aspergillus flavus* resulted in fold increases of 1.45 for vindoline, 3.29 for catharanthine, and 2.14 for ajmaline (Liang et al., 2018).

Plant roots can also form beneficial symbiotic interactions with endophytic bacteria and fungus. The process of establishing endophytic colonization can lead to an elicitation of plant defense responses (Yan et al., 2019). Twenty-seven fungal and bacterial endophytes were isolated from an alkaloid-rich *C. roseus* cultivar Dhawal. Two of the fungal isolates, a hyphomycete fungus *Curvularia* sp. which can form both beneficial and pathogenic plant interactions and *Choanephora infundibulifera* a pathogen, were shown to enhance serpentine content by 211.7–337.6% in a low alkaloid yielding *C. roseus* cultivar Prabal. Two other isolates, a fungus *Aspergillus japonicus* and a bacteria of *Pseudomonas* sp., increased the content of ajmalicine by 123.4–203.8% (Singh et al., 2020a).

Salicylic acid is a plant hormone involved in innate immunity (Ding and Ding, 2020). Foliar application of salicylic acid at concentrations of 0.1 mm and 0.01 mm resulted in increased transcription of 6 genes involved in the TIA biosynthetic pathway as measured with qRT-PCR. The genes examined were: chorismate mutase (CM), TDC, G10H, SLS, STR, D4H, and DAT (Soltani et al., 2020).

MeJA is a volatile phytohormone involved in the plant stress response to insect herbivory, and it is involved in some developmental pathways (Singh and Dwivedi, 2018). The phytohormone ethylene is also involved in plant defense and wounding response (Phukan et al., 2017). MeJA and ethylene signaling pathways have synergistic effects with regards to plant defense responses but can act antagonistically with regards to some developmental and wounding responses (Zhu, 2014). A transcriptomics study utilizing next generation sequencing showed that in roots, monoterpenoid biosynthesis genes respond specifically to MeJA, while phenolic biosynthesis genes respond specifically to ethylene in leaves. It was also found that TIA accumulation was strictly regulated by ERFs and BIS1 transcription factors. This work was also able to correlate the expression of specific ATP-binding cassette (ABC) transporters to TIA synthesis and specific transcription factors. In roots, ABC2 and ABC8 were co-expressed with ERFs. In leaves, ABC3 and the ABC transporter CrTPT2 showed co-expression with BIS1 which correlated with accumulation of vindoline. Overall, it was concluded that ethylene has a stronger effect than MeJA on TIA induction at both the transcriptional and metabolite level (Pan et al., 2018).

Metabolomic analysis of *C. roseus* leaves revealed that ethylene and MeJA control different metabolic networks to induce TIA biosynthesis. Ethylene regulates the 2-C-methyl-D-erythritol 4-phosphate (MEP) pathway through the enzyme, 1-deoxy-D-xylulose-5-phosphate synthase (DXS). MeJA regulates the acetate−mevalonate (MVA) pathway through the enzymes, Acetoacetyl-CoA thiolase (AACT) and Hydroxymethylglutaryl-CoA synthase (HMGS). The MEP and MVS pathways converge on isopentenyl pyrophosphate (IPP) which is a major intermediate in the biosynthesis of terpenoids. TIAs specifically upregulated by ethylene include ajmaline, 1,2-dihydrovomilenine, serpentine, 16-methoxytabersonine, *α*-3′,4′-anhydrovinblastine, and vinblastine. TIAs specifically upregulated by MeJA include strictosidine, strictosidine-aglycone, tabersonine, 16 hydroxytabersonine, deacetoxyvindoline, catharanthine, 16-methoxy-2,3-dihydro-3-hydroxytabersonine, and cathenamine (Zhang et al., 2018).

#### Environmental stresses and light elicitation

When *C. roseus* was subjected to drought stress tryptophan and tryptamine (Indole pathway) and serpentine and catharanthine (TIA Pathway) contents increased in both roots and leaves. Ajmalicine was found only in roots and showed a decrease with drought stress (Shabnam Akhtar et al., 2017). TIA levels in leaves, specifically tabersonine, catharanthine, vindoline, vinblastine and vincristine increase during the process of photomorphogenesis and peaked during cotyledon opening (Yu et al., 2018). Under UV-B light levels, there is an increase in TIA metabolite accumulation and a transcriptional increase in G10H, TDC, and STR in *C. roseus* hairy roots (Takshak and Agrawal, 2019).

#### Temperature effect on TIA production levels

When *C. roseus* were grown in pots in a circulating water bath system for two days at root-zone temperatures of 12°C, 25°C (control temperature), and 30°C, incubation at a temperature of 12°C greatly enhanced the root ajmalicine content (2.38 mg g − 1) almost 3 times higher than the 25°C control temperature. Catharanthine levels were also higher at 12°C compared to 25°C and 30°C. TDC and SGD gene expression in roots was significantly higher when grown in a root-zone temperature of 12°C (Malik et al., 2013). Higher bisindole alkaloid (vinblastine and vincristine) content in leaves was observed during summer, when the average temperature was ≥40°C. Whereas, the content of vindoline a monomeric TIA was higher in winter and spring. Also of interest, the monomeric TIAs were expressed at the highest levels in the top-level leaf-pairs, whereas the bisindole alkaloids were expressed at the highest levels in the middle-level leaf-pairs (Mall et al., 2019).

## Recent examples of metabolic engineering and synthetic biology approaches to increase TIA production in *Catharanthus roseus*

### Overexpression of biosynthetic enzymes

Attempts to improve TIA production in *C. roseus* have focused on genetically engineering *C. roseus* hairy roots with production using plant tissue culture techniques. Most studies either overexpressed key rate limiting TIA pathway enzymes and/or transcription factors regulating TIA pathways using *A. rhizogenes* to introduce DNA of interest into the genome. Common plant parts are often used to control overexpression of genes. Often results were a mix of increased and decreased metabolite concentrations.

Based on the results of feeding experiments examining TIA production in hairy root cultures, the enzyme TDC or STR were often overexpressed. Inducible promoters were often used because past constitutive expression studies presented problems due to high clonal variation in TIA production and/or high variability in expression levels (Ayora-Talavera et al., 2002). Overexpression of STR resulted in production of 200 mg/l of strictosidine and/or strictosidine-derived TIAs, including ajmalicine, catharanthine, serpentine, and tabersonine (Canel et al., 1998). Expression of DXS (part of the MEP pathway) in hairy roots from an inducible promoter resulted in an increase of ajmalicine by 67%, serpentine by 26%, and lochnericine by 49%, but there was a decrease in tabersonine by 66% and hörhammericine by 54%. Co-expression of DXS with G10H showed an increase in ajmalicine by 16%, lochnericine by 31% and tabersonine increased by 13% instead of the decrease that was seen when DXS was overexpressed alone. DXS and anthranilate synthase A subunit (ASA) co-expression showed an increase in hörhammericine by 30%, lochnericine by 27%, and tabersonine by 34% (Peebles et al., 2011). Over-expressing the two upstream pathway genes TDC and STR in transgenic *C. roseus* resulted in two-fold enhanced total alkaloid production with maximum nine-fold increase in vindoline and catharanthine, and five-fold increased vinblastine production. These lines recorded a maximum of 38-fold and 65-fold enhanced transcript levels of CrTDC and CrSTR genes, respectively (Sharma et al., 2018).

### Overexpression of transcription factors

Reports of TIA transcription factor overexpression in *C. roseus* hairy roots include overexpressing the AP2/ERF-domain transcription factor ORCA3 using a glucocorticoid inducible promoter along with Jasmonic Acid elicitation. Overexpressing ORCA3 resulted in an overexpression of transcripts for ZCT1 and ZCT2 which are TIA pathway repressors (Peebles et al., 2009). ORCA2 was overexpressed in *C. roseus* hairy roots using the constitutive cauliflower mosaic virus 35S promoter (CaMV-35S). The average content of catharanthine and vindoline was increased up to 2.03 and 3.67-fold versus control levels (Dong Hui et al., 2011). In contrast to ORCA3, overexpression of ORCA4 overexpression results in a dramatic increase of TIA accumulation in *C. roseus* hairy roots. Overexpression of CrMAPKK1 a previously uncharacterized MAP kinase shown in this work to act upstream of the ORCA gene cluster upregulated TIA pathways genes and increased TIA in *C. roseus* hairy roots (Paul et al., 2020).

CrWRKY1 is primarily expressed in roots and induced by jasmonate, gibberellic acid, and ethylene. Constitutive expression of CrWRKY1 using CaMV-35S resulted in an 7–9-fold increase in the TDC gene however transcript levels for the TIA pathway repressors ZCT1, ZCT2 and ZCT3 also increased, and there was a repression of TIA pathway activators ORCA2, ORCA3, and CrMYC2. Fusing the SRDX repressor domain to CrWRKY1 to convert CrWRKY1 from an activator to a repressor resulted in repression of TDC and the ZCTs but the up-regulation of ORCA3 and CrMYC2. Serpentine levels increased in 3 independent CrWRKY1 overexpression lines ca. 225–375 mg/gram of dry weight vs. ca 125 mg/gram in the empty vector controls, but catharanthine, ajmalicine, and tabersonine levels were near empty vector control line levels (Suttipanta et al., 2011).

One promising strategy when overexpressing transcription factors involved in TIA biosynthesis to increase TIA levels was to also overexpress a TIA enzyme (either indole/terpenoid precursor pathway or TIA pathway) which was not regulated by the transcription factor. Overexpressing ORCA3 in *C. roseus* hairy roots by itself resulted in no change in the total amount of TIAs measured. However, co-overexpression of ORCA3 and SGD resulted in a significant (*p* < 0.05) increase in serpentine by 44%, ajmalicine by 32%, catharanthine by 38%, tabersonine by 40%, lochnericine by 60% and hörhammericine by 56% (Sun and Peebles, 2016a).

### Other strategies for increasing TIA accumulation

A common strategy for improving production in microbial systems is to screen a large variety of variants to pick the most favorable one as the starting point to improve production. This strategy can also be applied to plants to look for varieties with higher TIA production. One study screened for *C. roseus* genotypes with a larger TIA accumulation. A TIA accumulation genotype (CIMAP866) which produced more vindoline was identified and characterized. It was comparatively analyzed with elite varieties, Dhawal and Nirmal. Its vindoline content in dry weight % (~0.20%) was significantly higher than that of Dhawal (~0.09%) and Nirmal (~0.04%; Mall et al., 2021). An ethylmethanesulfonate mutagenized *C. roseus* mutant line M2-1865 which accumulated vindoline pathway intermediates was identified in a screen. The mutation was mapped to the enzyme tabersonine 3-reductase (TR3; Figure 2). Mutation in this gene led to the stable formation and accumulation of high levels of tabersonine-2,3-epoxide and 16-methoxytabersonine-2,3-epoxide epoxides providing a dependable biological source for these two TIAs (Edge et al., 2018). These varieties have not been tested for their suitability as a hairy root culture.

One area largely neglected in improving TIA production is the engineering of TIA transporters. One exception is a studied that explored the overexpression of the catharanthine transporter, CrTPT2, in *C. roseus* hairy roots using a constitutive promoter. This study showed an increased catharanthine accumulation fivefold higher than that in control hairy roots, while other TIA accumulation levels were not affected. This demonstrates that removal of the TIAs from site of synthesis is crucial when trying to increase yields (Wang et al., 2019).

*Catharanthus roseus* hairy roots were batch cultivated in a statistically optimized nutrient medium. A mathematical model was developed using the observed batch kinetics data. Off-line simulations of the model extrapolated to fed-batch cultivation was used to identify suitable nutrient feeding strategies for the overproduction of ajmalicine. A 60% increase in ajmalicine and a 2.5-fold increase in volumetric yield was obtained by using a model predicted “increasing feed rate strategy” as opposed to batch cultivation. Further optimization using a statistically optimized mixture of elicitors (jasmonic acid, methyl jasmonate and KCl) resulting in a significantly high ajmalicine concentration of 123.2 ± 8.63 mg/l about 4 fold increase over the original batch cultivation (Thakore et al., 2015).

## Future prospective for TIA production in *Catharanthus roseus* hairy roots

The results of the TIA transcription factor overexpression studies show complex results where positive regulators of TIA biosynthesis often activate negative regulators of TIA biosynthesis. These results suggest that endogenous TIA production has evolved for short bursts of TIA production quickly followed by a shutdown. Efforts to engineer *C. roseus* hairy roots to sustainably produce TIAs will need to either engineer orthogonal biosynthetic pathways or utilize methodologies such as RNAi or CRISPR based genome editing to alleviate this negative feedback on endogenous pathways. As these recent research examples have shown, much progress has been made in optimizing TIA production in *C. roseus* hairy roots. To ultimately see improvements in TIA production in hairy roots, many factors will need to be considered such as genetic background, growth/incubation temperature, developmental stage, transportation of the product away from the site of production, transcriptional expression strategies (inducible or tissue specific promoters versus constitutive promoters), enzymes and/or transcription factors overexpress/knockdown, and use of bioreactors with mathematical modeling based optimization.

## Growth in bioreactors

For hairy root tissue culture to transition out of the lab into industrial production of products, one must be able to grow them at larger scales than a shake flask. There are many excellent reviews discussing scale up of hairy roots (Stiles and Liu, 2013; Jeong and Park, 2017; Mehrotra et al., 2018; Kareem et al., 2019; Valdiani et al., 2019). Here we will highlight the various bioreactor design configurations. The type of bioreactors used for hairy roots will differ substantially than those used for microbial cultivation or mammalian cell culture. The biggest difference is that an agitator should not be utilized for stirring due to the shear stress damage that agitation would cause to the hairy roots. Since agitation is primarily used to overcome mass transfer limitation of gas transport into the culture media, other solutions must be employed for hairy root cultivation. These solutions take two forms: liquid phase and gas phase cultivation.

### Liquid phase cultivation

Here the hairy roots are submerged in liquid media. Since mixing and mass transfer limitations are the key limiting process parameters for achieving optimal growth in these systems, there have been many different designs developed to overcome these limitations. The most used reactor in industrial biotechnology is the stirred tank reactor. The cylindrical vessel is usually composed of glass (small scale) or stainless steel (large scale) with a sparger located at the bottom of the tank to supply air to the culture and a shaft with an impeller to aide in mixing and mass transfer of oxygen to the growth media. Since oxygen transport from gas to liquid is limiting, the goal is to have small bubbles with longer residence times in the reactor. For microbial fermentation, this means that you stir the reactor at high rates to improve your oxygen transfer. As agitation speed increases, shear stress experienced by the cells also increases which damages hairy root tissue. This results in less productivity overall. One way to circumvent the shear stress is to protect the roots from the impeller. To do this, a mesh or support structure is utilized to physically separate the roots from the impeller. This helps to protect the hairy roots from damage.

Another way to protect the hairy roots from a propeller is to remove it and provide oxidation and mixing in another way. Bubble column reactors rely on gas bubbles introduced at the bottom of the reactor to provide mixing and gas exchange as they flow through the hairy root bed. These reactors sometimes have support structures for the hairy roots to grow on but not always. While this is a low-cost setup, gas bubbles can be trapped within the hairy roots; channeling around root clumps can occur; and dead zones of depleted oxygen and nutrients can reduce overall biomass production. Air lift reactors are similar to bubble columns except for a draft tube that improves liquid circulation throughout the reactor but often causes excessive foaming and requires more energy than a bubble column. Convective flow reactors utilize a two-vessel setup where one vessel is a stirred tank reactor that is utilized to oxygenate the culture media while the second vessel contains support structures to grow the hairy roots allowing for the oxygenated media to flow through the root bed.

One reactor type not used as often for hairy root cultivation is a wave bioreactor (Eibl and Eibl, 2008). These bioreactors were first developed for mammalian cell culture applications. These reactors are usually made from a disposable plastic bag that allows for laminar air flowrates. The reactor sits on a rocking platform that generates gentle waves that are used for mixing and oxygen transfer.

### Gas phase cultivation

Gas phase reactors are often a two-stage reactor system. The first stage operates as a liquid phase reactor to allow for dispersal and growth of hairy roots along a support structure. This avoids a labor-intensive set-up of manually dispersing the hairy roots on the support structure. The second stage often operates in one of two modes. One mode is a trickle bed where media is slowly trickled through the hairy root bed. This allows for abundant oxygen supply to the root culture, but one may have problems with channeling of the liquid through the bed limiting nutrients to some roots and with a viscous liquid film coating the roots creating a high mass transfer barrier. Another mode is a nutrient mist reactor where the media is supplied as a fine mist to the hairy root bed. This reactor type also offers abundant oxygen supply but requires high energy costs to operate.

## Conclusion: Future prospective in tapping the potential of hairy roots

There is a promising future for the use of hairy roots in plant product production. The ability to mine large genomic and transcriptomic data sets to identify missing/unknown product pathway enzymes or identify enzyme variants with higher activity will result in increased product yield. Using these databases to identify competing metabolic pathways or identifying negative feedback in product pathways and then engineering strategies to circumvent them is especially important when working with secondary metabolites such as terpenoid indole alkaloids. Genomic engineering with CRISPR technology is starting to be applied in the hairy root field with more papers published on using CRISPR in hairy roots (46 papers) in the last two years than in all previous years combined (32 papers; Kiryushkin et al., 2021). Bioproduction in designer or custom cell lines which have extensive genomic modifications has increased productivity in bacteria and yeast systems. We are now developing the tools to make the same types of custom modifications in plants and hairy roots is a system which lends itself to such modifications.

## Author contributions

KM and CP conceived of the paper idea and contributed to the writing and editing of this paper. All authors contributed to the article and approved the submitted version.

## Funding

This work was supported by the National Science Foundation Awards #1803437.

## Conflict of interest

The authors declare that the research was conducted in the absence of any commercial or financial relationships that could be construed as a potential conflict of interest.

## Publisher’s note

All claims expressed in this article are solely those of the authors and do not necessarily represent those of their affiliated organizations, or those of the publisher, the editors and the reviewers. Any product that may be evaluated in this article, or claim that may be made by its manufacturer, is not guaranteed or endorsed by the publisher.
